# Data on biodistribution and radiation absorbed dose profile of a novel ^64^Cu-labeled high affinity cell-specific peptide for positron emission tomography imaging of tumor vasculature

**DOI:** 10.1016/j.dib.2016.02.080

**Published:** 2016-03-04

**Authors:** Joseph R. Merrill, Krzysztof Krajewski, Hong Yuan, Jonathan E. Frank, David S. Lalush, Cam Patterson, Anka N. Veleva

**Affiliations:** a Biomedical Research Imaging Center, University of North Carolina, Chapel Hill, NC 27599, USA; b Department of Biochemistry and Biophysics, University of North Carolina, Chapel Hill, NC 27599, USA; c Department of Biomedical Engineering, North Carolina State University, Raleigh, NC 27695, USA; d NewYork Presbyterian Hospital, Weill Cornell Medical Center, New York, NY 10065, USA; e Department of Chemical and Biomolecular Engineering, North Carolina State University, Raleigh, NC 27695, USA

**Keywords:** Molecular imaging, Tumor angiogenesis, Radiation absorbed dose, Diagnostic radiopharmaceuticals

## Abstract

New peptide-based diagnostic and therapeutic approaches hold promise for highly selective targeting of cancer leading to more precise and effective diagnostic and therapeutic modalities. An important feature of these approaches is to reach the tumor tissue while limiting or minimizing the dose to normal organs. In this context, efforts to design and engineer materials with optimal in vivo targeting and clearance properties are important. This Data In Brief article reports on biodistribution and radiation absorbed dose profile of a novel high affinity radiopeptide specific for bone marrow-derived tumor vasculature. Background information on the design, preparation, and in vivo characterization of this peptide-based targeted radiodiagnostic is described in the article “Synthesis and comparative evaluation of novel 64Cu-labeled high affinity cell-specific peptides for positron emission tomography of tumor vasculature” (Merrill et al., 2016) [1]. Here we report biodistribution measurements in mice and calculate the radiation absorbed doses to normal organs using a modified Medical Internal Radiation Dosimetry (MIRD) methodology that accounts for physical and geometric factors and cross-organ beta doses.

**Specifications Table**

**Table:** 

Subject area	*Chemistry in radiology*
More specific subject area	*Materials Science*
Type of data	*Tables*
How data was acquired	*Direct collection of tissues from mice at different time-points post-injection*
Data format	*Analyzed data*
Experimental factors	*Novel radiopeptide targeting tumor vasculature was administered intravenously into mice*
Experimental features	*Biodistribution data were analyzed using more precise method accounting for the small size of the murine organs and for the type of radionuclide emissions*
Data source location	*USA*
Data accessibility	*Data is provided within this article*

**Value of the data**

• The data reported here demonstrate how mouse tissue distribution measurements can be used to determine radiation absorbed dose to normal mouse tissues and organs.
• Using this improved dosimetric approach that incorporates cross-organ beta doses permits refined and more accurate estimates of the radiation absorbed dose to normal tissues.
• Accurate absorbed dose estimates which account for the size (mass and dimensions) of the murine organs or lesions as well as for the decay characteristics of the radionuclide are essential in evaluating novel radiodiagnostic and radiotherapeutic compounds.
• Cross-organ contributions may be very important for understanding differences in biologic effects in tumors and micro metastasis in different organs.
• The data presented here can be of value to material scientists and engineers to more accurately evaluate radiation safety profiles of new radiodiagnostic and radiopharmaceutic compositions in mice.

## 1 Data

Previous reports have shown that the high affinity, high specificity FHT-peptide selected by screening of a bacteriophage library [2], [3], when labeled with the positron emission tomography radionuclide, copper-64, ^64^Cu, can specifically image bone marrow-derived tumor vasculature [1], [4]. In this Data in Brief article we provide data on biodistribution of this radiopeptide at 1 h, 4 h, and 24 h post- injection in a subcutaneous Lewis lung carcinoma tumor model (Table 1). From the biodistribution data, using a geometric model that accounts for the small size of the murine organs [5] and for the decay characteristics of the ^64^Cu radionuclide [6], we have determined the specific accumulated activity in mouse organs over time (Table 2) and we have calculated radiation absorbed dose in the liver, spleen, kidney, lungs, heart, stomach, small and large intestine, thyroid, pancreas, and murine carcass (Table 3).

**Table 1:** Biodistribution of ^64^Cu-labeled FHT-peptide in C57BL/6 mice. Values are presented as mean±SD (%ID/g) (*n*=3).

Organ		Time post-injection							
	1 h			4 h			24 h		
Liver	1.61±0.21			1.50±0.67			0.86±0.34		
Spleen	0.57±0.14			0.96±0.54			0.37±0.06		
Kidney	5.28±0.82			3.37±1.77			1.12±0.48		
Lungs	1.68±0.58			1.18±0.54			0.56±0.27		
Heart	0.31±0.14			0.39±0.15			0.30±0.08		
Stomach	0.41±0.07			1.73±0.48			0.34±0.06		
Small Intestine	1.10±0.86			0.96±0.71			0.52±0.13		
Large Intestine	0.66±0.44			1.03±0.48			0.53±0.17		
Thyroid	0.20±0.06			0.50±0.14			0.13±0.06		
Pancreas	0.55±0.25			0.25±0.09			0.26±0.07		
Carcass	0.10±0.02			0.12±0.10			0.04±0.02

**Table 2:** Specific accumulated activity in mouse organs over time. Values in MBq s/g per MBq injected dose, presented as mean±SD (*n*=3).

Organ		Time interval post-injection											Total activity		
	0–1 h			1–4 h			4–24 h			24 h-infinity			MBq s/g MBq		
Liver	56.8±6.8			147.6±31.5			617.3±209.5			335.0±129.6			1156.7±248.4		
Spleen	18.2±4.8			71.3±24.7			357.6±158.2			143.0±19.1			590.2±161.4		
Kidney	193.6±24.8			416.8±87.6			1214.6±512.2			431.4±175.2			2256.5±557.3		
Lungs	60.9±16.6			137.3±37.6			461.7±164.6			215.9±100.7			875.8±197.2		
Heart	10.4±3.9			32.9±9.4			178.1±45.2			118.1±29.2			339.5±54.8		
Stomach	8.0±3.0			96.2±21.0			573.5±138.3			132.4±18.2			810.0±141.1		
Small Intestine	39.0±24.6			97.8±53.4			387.9±205.0			201.8±46.8			726.5±218.4		
Large Intestine	21.4±12.8			78.6±31.1			410.9±146.0			206.6±59.8			717.5±161.3		
Thyroid	5.5±1.7			32.3±6.9			173.2±43.6			48.8±21.6			259.8±49.2		
Pancreas	20.6±7.2			38.9±13.6			128.3±28.5			102.4±23.2			290.2±39.8		
Carcass	3.3±0.8			10.1±4.5			50.6±34.1			29.5±32.9			93.4±47.7

**Table 3:** Radiation absorbed doses in C57Bl6 mice calculated using biodistribution data and the cross-organ mouse model [6]. Values in mGy per MBq injected dose.

Target organ, k	Source organ, *h*											Total dose
	Liver	Spleen	Kidney	Lungs	Heart	Stomach	Small intestine	Large intestine	Thyroid	Pancreas	Carcass	mGy/MBq
Liver	21.01	0.00	0.23	0.05	0.01	0.13	0.02	0.00	0.00	0.64	0.03	21.48
Spleen	0.00	9.28	0.96	0.00	0.00	0.95	0.00	0.00	0.00	0.15	0.00	11.83
Kidney	0.28	0.15	38.72	0.00	0.00	0.27	0.10	0.02	0.00	0.00	0.00	39.68
Lungs	1.13	0.00	0.00	13.81	0.18	0.11	0.00	0.00	0.00	0.00	0.13	15.36
Heart	0.21	0.00	0.00	1.28	5.82	0.00	0.00	0.00	0.00	0.06	0.00	7.30
Stomach	0.18	0.24	0.31	0.05	0.00	14.51	0.06	0.00	0.00	0.00	0.00	15.40
Small Intestine	0.27	0.00	0.41	0.00	0.00	0.08	13.18	0.37	0.00	0.00	0.07	14.38
Large Intestine	0.14	0.00	0.26	0.00	0.00	0.02	0.33	12.75	0.00	0.00	0.00	13.51
Thyroid	0.00	0.00	0.00	0.00	0.00	0.00	0.00	0.00	3.48	0.00	0.29	3.77
Pancreas	0.00	0.45	0.83	0.00	0.00	0.23	0.00	0.00	0.00	4.37	0.00	5.89
Carcass	0.04	0.00	0.01	0.03	0.00	0.00	0.00	0.00	0.01	0.00	1.76	1.90

## 2 Experimental design, materials and methods

The animal protocols were approved by the University of North Carolina at Chapel Hill Institutional Animal Care and Use Committee.

### 2.1 Biodistribution in tumor bearing C57BL/6 mice

Approximately 0.74 MBq of ^64^Cu-labeled peptide in a total volume of 150 μl of saline was injected under anesthesia intravenously via a catheter in the tail vein in Lewis lung carcinoma (LLC) tumor-bearing C57BL/6 mice (*n*=3 per time point) as described in Ref. [1]. At 1 h, 4 h, or 24 h post-injection mice were sacrificed under anesthesia and liver, spleen, kidney, lungs, heart, stomach, small and large intestines, thyroid, pancreas, and muscle were harvested. The organs were weighted and their radioactivity was measured in an automated gamma counter (WIZARD2, Perkin Elmer Life Sciences, Gaithersburg, MD). Radioactivity concentrations were calibrated, decay-corrected, and expressed as percentage of injected dose per gram tissue (%ID/g). The percentage of injected dose per gram carcass was approximated from radioactivity in a sample of muscle. The biodistribution data at different time points post injection are presented in Table 1.

### 2.2 Dosimetry calculations

The method for calculating the dose from internal beta emitters in mice was developed for ^90^Y by Hui et al. [5] and extended to other radionuclides, including ^64^Cu, by Miller et al. [6]. This method includes cross-organ contributions from beta particles and ignores the dose from photons which are more likely to completely escape the mouse. The absorbed dose, D, to a target organ, *k*, from a source organ, *h*, is given by:

$$D\left(r_{k}\leftarrow r_{h}\right)=K{\bar{E}}_{\beta }\varphi_{\beta }\left(r_{k}\leftarrow r_{h}\right)\left(\frac{{\tilde{A}}_{h}}{m_{h}}\right)\left(\frac{m_{h}}{m_{k}}\right)$$

where *K* is a unit conversion constant, ${\bar{E}}_{\beta }$ is the average energy of emitted beta particles (0.121 MeV for ^64^Cu), $\varphi_{\beta }\left(r_{k}\leftarrow r_{h}\right)$ is the fraction of energy emitted in source organ *h* that is absorbed in target organ *k*, $\frac{{\tilde{A}}_{h}}{m_{h}}$ is the specific accumulated activity in the source organ *h*, and $m_{k}$ is the mass of the target organ *k*. Absorbed fractions in mouse organs for ^64^Cu are taken from Ref. [6].

Accumulated activity is found from the gamma counting biodistribution data collected at 1, 4 and 24 h post-injection converted to specific activity in Bq/g and normalized to an injected dose of 1 MBq. These values are plotted with respect to time of sacrifice to create a time-activity curve and the accumulated activity is found by integrating this curve. Integration was performed by the trapezoidal method up to the 24 h time point. Activity trapped in an organ after 24 h was considered to be cleared only by the physical decay of ^64^Cu with a half-life of 12.701 h. The accumulated activity from this period was found by integrating the exponential decay to infinity:

$${\tilde{A}}_{h,t>24h}=A_{24h}\int_{t=24h}^{\infty }\exp\left(-\frac{0.693}{12.701h}\left(t-24h\right)\right)\mathrm{dt}$$

The specific accumulated activity in each organ for each time interval measured and over all time is summarized in Table 2. The accumulated activity contained in each source organ is found by multiplying the accumulated activity per gram by the average mass of the organ.

The source-to-target doses for each MBq of injected activity are summarized in Table 3. For each target organ, the total dose is found by summing the contributions from all source organs:

$$D\left(r_{k}\leftarrow r_{h}\right)=K{\bar{E}}_{\beta }\sum_{h}\varphi_{\beta }\left(r_{k}\leftarrow r_{h}\right)\left(\frac{{\tilde{A}}_{h}}{m_{h}}\right)\left(\frac{m_{h}}{m_{k}}\right)$$

The final column of Table 3 displays these total target organ absorbed doses in mGy per MBq of injected activity.
