# Supplementary material for: Data on biodistribution and radiation absorbed dose profile of a novel 64Cu-labeled high affinity cell-specific peptide for positron emission tomography imaging of tumor vasculature
Source: Data Brief. 2016 Mar 4;7:480–4. doi: 10.1016/j.dib.2016.02.080 (PMC4792855; doi:10.1016/j.dib.2016.02.080)
Supplement: Supplementary file 1 — Supplementary material [file mmc1.pdf]

Conflict of Interest:

NONE
